# [2,7-Dimeth­oxy-8-(4-propyl­benzo­yl)naphthalen-1-yl](4-propyl­phen­yl)methanone

**DOI:** 10.1107/S1600536812033582

**Published:** 2012-07-28

**Authors:** Kosuke Sasagawa, Daichi Hijikata, Rei Sakamoto, Akiko Okamoto, Noriyuki Yonezawa

**Affiliations:** a Department of Organic and Polymer Materials Chemistry, Tokyo University of Agriculture & Technology, Koganei, Tokyo 184-8588, Japan

## Abstract

In the title compound, C_32_H_32_O_4_, the 4-propyl­benzoyl groups at the 1- and 8-positions of the naphthalene ring system are aligned almost anti­parallel, and their benzene rings make a dihedral angle of 8.64 (10)°. The dihedral angles between the naphthalene ring system and the benzene rings are 69.37 (8) and 69.45 (8)°. In the crystal, C—H⋯O inter­actions link adjacent mol­ecules *via* their aroyl groups.

## Related literature

For the formation reaction of aroylated naphthalene compounds *via* electrophilic aromatic substitution of naphthalene derivatives, see: Okamoto & Yonezawa (2009 ▶); Okamoto *et al.* (2011 ▶). For the structures of closely related compounds, see: Hijikata *et al.* (2010 ▶); Muto *et al.* (2010 ▶); Sasagawa, Hijikata *et al.* (2011) ▶; Sasagawa, Muto *et al.* (2011 ▶); Sasagawa *et al.* (2012 ▶).
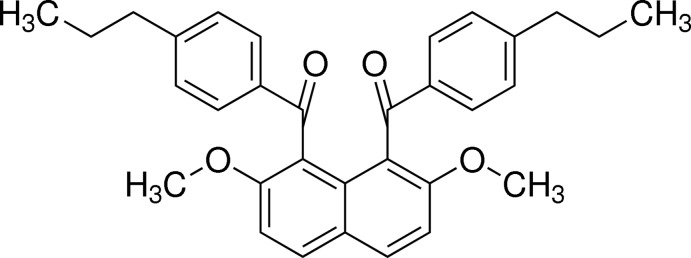


## Experimental

### 

#### Crystal data

C_32_H_32_O_4_
*M* *_r_* = 480.58
Monoclinic, 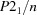

*a* = 18.1224 (3) Å
*b* = 7.91914 (14) Å
*c* = 19.7355 (4) Å
β = 113.502 (1)°
*V* = 2597.37 (8) Å^3^
*Z* = 4
Cu *K*α radiation
μ = 0.63 mm^−1^
*T* = 193 K
0.40 × 0.30 × 0.05 mm

#### Data collection

Rigaku R-AXIS RAPID diffractometer
Absorption correction: numerical (*NUMABS*; Higashi, 1999 ▶) *T*_min_ = 0.786, *T*_max_ = 0.969
45013 measured reflections
4752 independent reflections
3258 reflections with *I* > 2σ(*I*)
*R*_int_ = 0.047

#### Refinement

*R*[*F* ^2^ > 2σ(*F* ^2^)] = 0.048
*wR*(*F* ^2^) = 0.152
*S* = 1.10
4752 reflections
330 parameters
H-atom parameters constrained
Δρ_max_ = 0.22 e Å^−3^
Δρ_min_ = −0.26 e Å^−3^

### 

Data collection: *RAPID-AUTO* (Rigaku, 1998 ▶); cell refinement: *RAPID-AUTO*; data reduction: *RAPID-AUTO*; program(s) used to solve structure: *Il Milione* (Burla *et al.*, 2007) ▶; program(s) used to refine structure: *SHELXL97* (Sheldrick, 2008 ▶); molecular graphics: *CrystalStructure* (Rigaku, 2010 ▶); software used to prepare material for publication: *CrystalStructure*.

## Supplementary Material

Crystal structure: contains datablock(s) I, global. DOI: 10.1107/S1600536812033582/hb6909sup1.cif

Structure factors: contains datablock(s) I. DOI: 10.1107/S1600536812033582/hb6909Isup2.hkl

Supplementary material file. DOI: 10.1107/S1600536812033582/hb6909Isup3.cml

Additional supplementary materials:  crystallographic information; 3D view; checkCIF report

## Figures and Tables

**Table 1:** Hydrogen-bond geometry (Å, °)

*D*—H⋯*A*	*D*—H	H⋯*A*	*D*⋯*A*	*D*—H⋯*A*
C13—H13⋯O3^i^	0.95	2.41	3.342 (3)	168
C24—H24⋯O4^ii^	0.95	2.45	3.390 (3)	170

Symmetry codes: (i) 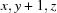; (ii) 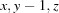.

**Fig. 1.**
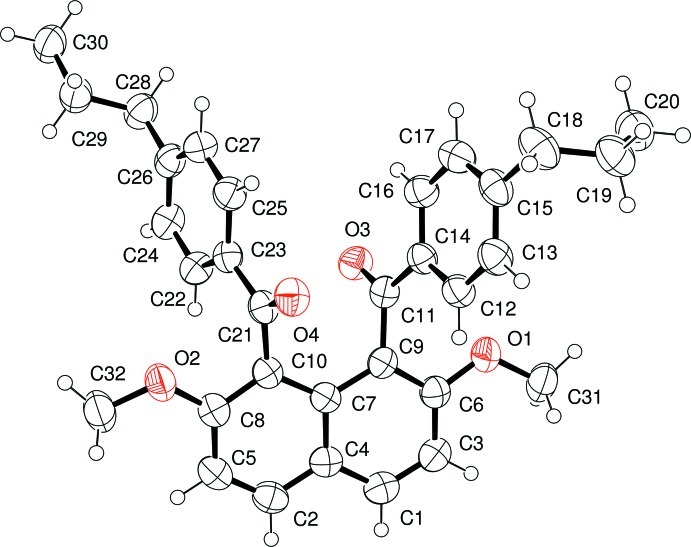
Molecular structure with displacement ellipsoids drawn at the 50% probability level.

**Fig. 2.**
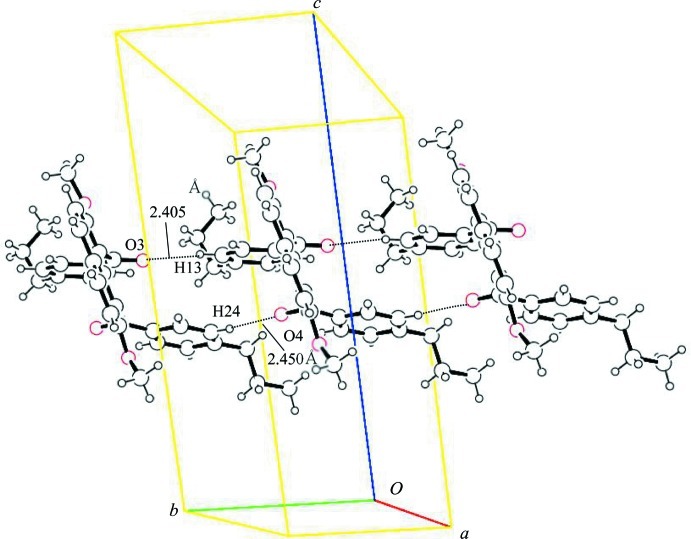
Intermolecular C—H···O interactions between H13 and O3 [symmetrycode: x, y + 1, z] and between H24 and O4 [symmetry code: x, y - 1, z] along the b axis (dashed lines).

## supplementary crystallographic information

### Comment

In the course of our study on selective electrophilic aromatic aroylation of the naphthalene ring core, 1,8-diaroylnaphthalene compounds have proved to be formed regioselectively by the aid of a suitable acidic mediator (Okamoto & Yonezawa, 2009, Okamoto *et al.*, 2011). Recently, we have reported the X-ray crystal structures of 1,8-diaroylated 2,7-dimethoxynaphthalene derivatives such as [2,7-dimethoxy-8-(4-methylbenzoyl)-1-naphthyl](4-methylphenyl)methanone [1,8-bis(4-methylbenzoyl)-2,7-dimethoxynaphthalene] (Muto *et al.*, 2010), {8-[4-(bromomethyl)benzoyl]-2,7-dimethoxynaphthalen-1-yl}[4-(bromomethyl)phenyl]methanone [1,8-bis(4-bromomethylbenzoyl)-2,7-dimethoxynaphthalene] (Sasagawa, Hijikata *et al.*, 2011), {8-[4-(butoxy)benzoyl]-2,7-dimethoxynaphthalen-1-yl}[4-(butoxy)phenyl]methanone [1,8-bis(4-butoxylbenzoyl)-2,7-dimethoxynaphthalene] (Sasagawa, Muto *et al.*, 2011), and 4-{[8-(4-acetyloxybenzoyl)-2,7-dimethoxynaphthalen-1-yl]carbonyl}phenyl acetate [1,8-bis(4-acetoxybenzoyl)-2,7-dimethoxynaphthalene] (Sasagawa *et al.*, 2012). The aroyl groups in these compounds are almost perpendicularly attached to the naphthalene rings and oriented in opposite directions (*anti*-orientation). Moreover, we have also shown that the aroyl groups of 2,7-dimethoxy-1,8-bis(4-phenoxybenzoyl)naphthalene (Hijikata *et al.*, 2010) are oriented in same direction (*syn*-orientation). As part of our ongoing studies on the molecular structures of these kinds of homologous molecules, the X-ray crystal structure of the title compound, 1,8-diaroylated naphthalene bearing propyl groups, is discussed in this article.

The molecular structure of the title compound is displayed in Fig 1. Two 4-propylbenzoyl groups are situated in *anti*-orientation. The dihedral angle between the best planes of the two phenyl rings is 8.64 (10) °. The dihedral angles between the best planes of the 4-propylphenyl rings and the naphthalene ring are 69.37 (8) and 69.45 (8) °.

Ketonic carbonyl moieties (C11, O3; C21, O4), carbon atoms (C18; C28) of propyl groups and benzene ring are lie on the same plane [torsion angles O3—C11—C14—C12 = -179.58 (18)°, C18—C15—C17—C16 = 178.9 (2)°; O4—C21—C23—C25 = -178.18 (19)°, C28—C26—C27—C25 = -179.5 (2)°].

In the molecular packing, two types of C—H···O interactions between carbonyl oxygen atom and hydrogen atom of the benzene ring are observed (Fig. 2).

### Experimental

To a 50 ml flask, 4-propylbenzoic acid (722 mg, 4.4 mmol), phosphorus pentoxide–methanesulfonic acid mixture (P_2_O_5_–MsOH [1/10 *w*/*w*]; 8.8 ml) were placed and stirred at 333 K. To the solution thus obtained, 2,7-dimethoxynaphthalene (376 mg, 2.0 mmol) was added. After the reaction mixture was stirred at 333 K for 1.5 h, it was poured into ice-cold water (10 ml). The aqueous layer was extracted with CHCl_3_ (10 ml × 3). The combined extracts were washed with 2 *M* aqueous NaOH followed by washing with brine. The organic layers thus obtained were dried over anhydrous MgSO_4_. The solvent was removed under reduced pressure to give cake. The crude product was purified by recrystallization from methanol (54% yield). Colorless platelet single crystals were obtained by repeated crystallization from ethanol solution.

^1^H-NMR δ (300 MHz, CDCl_3_): 0.95 (6*H*, t, *J* = 7.2 Hz), 1.65 (4*H*, q, *J* = 7.2 Hz), 2.60 (4*H*, t, *J* =7.2 Hz), 3.69 (6*H*, s), 7.11 (4*H*, d, *J* = 7.5 Hz), 7.21 (2*H*, d, *J* = 8.7 Hz), 7.58 (4*H*, d, *J* = 7.5 Hz), 7.94 (2*H*, d, *J* = 9.0 Hz) p.p.m.

^13^C-NMR δ (75 MHz, CDCl_3_): 13.9, 24.0, 38.2, 56.5, 111.3, 121.9, 125.5, 128.0, 129.2, 129.6, 131.8, 136.5, 147.8, 156.1, 196.2 p.p.m.

IR (KBr): 2952 (CH_3_), 2927 (CH_2_), 1656 (C=O), 1605, 1510, 1460 (Ar) cm^-1^

HRMS (m/*z*): [*M*+H]^+^ calcd. for C_32_H_33_O_4_,481.2379, found, 481.2421.

m.p. = 447.1—448.9 K

### Refinement

All H atoms were found in a difference map and were subsequently refined as riding atoms, with C—H = 0.95 (aromatic) and 0.98 (methyl) Å, and with *U*_ĩso_(H) = 1.2 *U*_eq_(C).

### Crystal data

**Table:**

C_32_H_32_O_4_	*F*(000) = 1024
*M**_r_* = 480.58	*D*_x_ = 1.229 Mg m^−^^3^
Monoclinic, *P*2_1_/*n*	Melting point = 448.9–447.1 K
Hall symbol: -P 2yn	Cu *K*α radiation, λ = 1.54187 Å
*a* = 18.1224 (3) Å	Cell parameters from 25236 reflections
*b* = 7.91914 (14) Å	θ = 4.3–68.2°
*c* = 19.7355 (4) Å	µ = 0.63 mm^−^^1^
β = 113.502 (1)°	*T* = 193 K
*V* = 2597.37 (8) Å^3^	Platelet, colorless
*Z* = 4	0.40 × 0.30 × 0.05 mm

### Data collection

**Table:**

Rigaku R-AXIS RAPID diffractometer	4752 independent reflections
Radiation source: rotating anode	3258 reflections with *I* > 2σ(*I*)
Graphite monochromator	*R*_int_ = 0.047
Detector resolution: 10.000 pixels mm^-1^	θ_max_ = 68.2°, θ_min_ = 4.3°
ω scans	*h* = −21→21
Absorption correction: numerical (*NUMABS*; Higashi, 1999)	*k* = −9→9
*T*_min_ = 0.786, *T*_max_ = 0.969	*l* = −23→23
45013 measured reflections	

### Refinement

**Table:**

Refinement on *F*^2^	Secondary atom site location: difference Fourier map
Least-squares matrix: full	Hydrogen site location: inferred from neighbouring sites
*R*[*F*^2^ > 2σ(*F*^2^)] = 0.048	H-atom parameters constrained
*wR*(*F*^2^) = 0.152	*w* = 1/[σ^2^(*F*_o_^2^) + (0.0684*P*)^2^ + 0.7538*P*] where *P* = (*F*_o_^2^ + 2*F*_c_^2^)/3
*S* = 1.10	(Δ/σ)_max_ < 0.001
4752 reflections	Δρ_max_ = 0.22 e Å^−^^3^
330 parameters	Δρ_min_ = −0.26 e Å^−^^3^
0 restraints	Extinction correction: *SHELXL97* (Sheldrick, 2008), Fc^*^=kFc[1+0.001xFc^2^λ^3^/sin(2θ)]^-1/4^
Primary atom site location: structure-invariant direct methods	Extinction coefficient: 0.0024 (2)

### Special details

**Table:**

Geometry. All e.s.d.'s (except the e.s.d. in the dihedral angle between two l.s. planes) are estimated using the full covariance matrix. The cell e.s.d.'s are taken into account individually in the estimation of e.s.d.'s in distances, angles and torsion angles; correlations between e.s.d.'s in cell parameters are only used when they are defined by crystal symmetry. An approximate (isotropic) treatment of cell e.s.d.'s is used for estimating e.s.d.'s involving l.s. planes.
Refinement. Refinement of *F*^2^ against ALL reflections. The weighted *R*-factor *wR* and goodness of fit *S* are based on *F*^2^, conventional *R*-factors *R* are based on *F*, with *F* set to zero for negative *F*^2^. The threshold expression of *F*^2^ > σ(*F*^2^) is used only for calculating *R*-factors(gt) *etc*. and is not relevant to the choice of reflections for refinement. *R*-factors based on *F*^2^ are statistically about twice as large as those based on *F*, and *R*- factors based on ALL data will be even larger.

### Fractional atomic coordinates and isotropic or equivalent isotropic displacement parameters (Å^2^)

**Table:**

	*x*	*y*	*z*	*U*_iso_*/*U*_eq_	
O1	0.24425 (9)	0.4558 (2)	0.71149 (8)	0.0569 (4)	
O2	0.01824 (9)	0.1904 (2)	0.33799 (8)	0.0610 (4)	
O3	0.27655 (8)	0.20263 (18)	0.58444 (8)	0.0532 (4)	
O4	0.17938 (9)	0.44541 (18)	0.42987 (8)	0.0542 (4)	
C1	0.03056 (14)	0.3547 (3)	0.61602 (12)	0.0540 (5)	
H1	−0.0172	0.3500	0.6250	0.065*	
C2	−0.04538 (13)	0.2496 (3)	0.49034 (13)	0.0535 (5)	
H2	−0.0924	0.2435	0.5005	0.064*	
C3	0.10007 (13)	0.4085 (3)	0.67087 (13)	0.0548 (5)	
H3	0.1008	0.4425	0.7173	0.066*	
C4	0.02756 (12)	0.3058 (3)	0.54627 (12)	0.0467 (5)	
C5	−0.04981 (13)	0.2041 (3)	0.42247 (13)	0.0553 (6)	
H5	−0.0989	0.1634	0.3860	0.066*	
C6	0.17079 (12)	0.4131 (3)	0.65775 (11)	0.0467 (5)	
C7	0.09867 (11)	0.3134 (2)	0.53196 (11)	0.0422 (5)	
C8	0.01909 (12)	0.2179 (3)	0.40692 (12)	0.0499 (5)	
C9	0.17178 (12)	0.3663 (2)	0.59093 (10)	0.0421 (5)	
C10	0.09209 (12)	0.2697 (2)	0.45953 (11)	0.0436 (5)	
C11	0.25296 (12)	0.3465 (3)	0.58720 (10)	0.0434 (5)	
C12	0.27818 (12)	0.6589 (3)	0.59160 (11)	0.0478 (5)	
H12	0.2277	0.6783	0.5944	0.057*	
C13	0.32583 (13)	0.7945 (3)	0.59118 (12)	0.0534 (5)	
H13	0.3076	0.9060	0.5935	0.064*	
C14	0.30310 (11)	0.4946 (2)	0.58802 (10)	0.0423 (5)	
C15	0.40005 (13)	0.7706 (3)	0.58743 (11)	0.0518 (5)	
C16	0.37687 (12)	0.4698 (3)	0.58295 (11)	0.0507 (5)	
H16	0.3947	0.3584	0.5797	0.061*	
C17	0.42412 (13)	0.6057 (3)	0.58265 (12)	0.0551 (6)	
H17	0.4742	0.5866	0.5791	0.066*	
C18	0.45184 (15)	0.9209 (3)	0.58852 (13)	0.0658 (7)	
H18A	0.4195	1.0017	0.5498	0.079*	
H18B	0.4971	0.8825	0.5764	0.079*	
C19	0.48555 (15)	1.0115 (3)	0.66281 (14)	0.0670 (7)	
H19A	0.5152	1.1129	0.6584	0.080*	
H19B	0.4401	1.0500	0.6747	0.080*	
C20	0.54096 (15)	0.9046 (4)	0.72574 (14)	0.0726 (7)	
H20A	0.5112	0.8074	0.7327	0.087*	
H20B	0.5618	0.9721	0.7711	0.087*	
H20C	0.5859	0.8648	0.7143	0.087*	
C21	0.15938 (12)	0.2993 (3)	0.43450 (11)	0.0445 (5)	
C22	0.17742 (13)	−0.0111 (3)	0.42097 (11)	0.0499 (5)	
H22	0.1315	−0.0332	0.4315	0.060*	
C23	0.20091 (12)	0.1545 (2)	0.41792 (10)	0.0436 (5)	
C24	0.21995 (14)	−0.1447 (3)	0.40895 (12)	0.0546 (6)	
H24	0.2023	−0.2570	0.4104	0.065*	
C25	0.26860 (13)	0.1837 (3)	0.40237 (12)	0.0518 (5)	
H25	0.2855	0.2960	0.3995	0.062*	
C26	0.28793 (13)	−0.1169 (3)	0.39483 (11)	0.0521 (5)	
C27	0.31099 (13)	0.0493 (3)	0.39115 (12)	0.0550 (6)	
H27	0.3569	0.0709	0.3807	0.066*	
C28	0.33506 (15)	−0.2620 (3)	0.38310 (12)	0.0611 (6)	
H28A	0.3271	−0.3622	0.4094	0.073*	
H28B	0.3930	−0.2330	0.4053	0.073*	
C29	0.31187 (15)	−0.3071 (3)	0.30285 (13)	0.0644 (6)	
H29A	0.3181	−0.2062	0.2760	0.077*	
H29B	0.2545	−0.3408	0.2809	0.077*	
C30	0.36266 (15)	−0.4497 (3)	0.29291 (14)	0.0688 (7)	
H30A	0.4194	−0.4164	0.3139	0.083*	
H30B	0.3457	−0.4739	0.2401	0.083*	
H30C	0.3556	−0.5510	0.3182	0.083*	
C31	0.24686 (15)	0.5212 (3)	0.77932 (12)	0.0656 (7)	
H31A	0.2307	0.4331	0.8055	0.079*	
H31B	0.2100	0.6173	0.7695	0.079*	
H31C	0.3017	0.5583	0.8098	0.079*	
C32	−0.04718 (15)	0.0998 (4)	0.28517 (13)	0.0715 (7)	
H32A	−0.0550	−0.0062	0.3071	0.086*	
H32B	−0.0355	0.0751	0.2418	0.086*	
H32C	−0.0962	0.1682	0.2702	0.086*	

### Atomic displacement parameters (Å^2^)

**Table:**

	*U*^11^	*U*^22^	*U*^33^	*U*^12^	*U*^13^	*U*^23^
O1	0.0583 (9)	0.0656 (10)	0.0449 (8)	−0.0052 (7)	0.0187 (7)	−0.0098 (7)
O2	0.0540 (9)	0.0761 (11)	0.0463 (9)	−0.0106 (8)	0.0131 (7)	−0.0089 (7)
O3	0.0574 (9)	0.0405 (8)	0.0617 (9)	0.0069 (7)	0.0237 (7)	0.0021 (7)
O4	0.0629 (9)	0.0414 (9)	0.0585 (9)	−0.0071 (7)	0.0244 (7)	0.0017 (7)
C1	0.0569 (13)	0.0492 (13)	0.0615 (14)	0.0022 (10)	0.0296 (11)	0.0040 (10)
C2	0.0476 (12)	0.0511 (13)	0.0618 (14)	−0.0006 (10)	0.0220 (10)	0.0066 (10)
C3	0.0626 (14)	0.0517 (13)	0.0547 (13)	−0.0001 (11)	0.0285 (11)	−0.0031 (10)
C4	0.0485 (11)	0.0405 (11)	0.0531 (12)	0.0021 (9)	0.0224 (10)	0.0044 (9)
C5	0.0448 (11)	0.0547 (14)	0.0597 (14)	−0.0039 (10)	0.0137 (10)	0.0014 (11)
C6	0.0497 (11)	0.0410 (11)	0.0480 (11)	0.0003 (9)	0.0182 (9)	0.0004 (9)
C7	0.0427 (10)	0.0352 (11)	0.0476 (11)	0.0003 (8)	0.0166 (9)	0.0030 (8)
C8	0.0491 (12)	0.0489 (12)	0.0486 (12)	−0.0028 (10)	0.0163 (10)	0.0002 (9)
C9	0.0489 (11)	0.0343 (10)	0.0439 (10)	0.0009 (8)	0.0195 (9)	0.0016 (8)
C10	0.0454 (11)	0.0377 (11)	0.0458 (11)	−0.0014 (8)	0.0163 (9)	0.0016 (9)
C11	0.0481 (11)	0.0395 (11)	0.0398 (10)	0.0039 (9)	0.0145 (9)	0.0018 (8)
C12	0.0438 (11)	0.0430 (12)	0.0525 (12)	0.0015 (9)	0.0148 (9)	0.0006 (9)
C13	0.0523 (12)	0.0440 (12)	0.0600 (13)	−0.0038 (10)	0.0182 (10)	0.0002 (10)
C14	0.0404 (10)	0.0414 (11)	0.0406 (10)	0.0011 (8)	0.0113 (8)	0.0025 (8)
C15	0.0504 (12)	0.0553 (14)	0.0439 (11)	−0.0089 (10)	0.0125 (9)	0.0045 (10)
C16	0.0482 (12)	0.0520 (13)	0.0504 (12)	0.0060 (10)	0.0180 (9)	0.0043 (10)
C17	0.0440 (11)	0.0649 (15)	0.0549 (13)	−0.0014 (10)	0.0182 (10)	0.0059 (11)
C18	0.0593 (14)	0.0668 (16)	0.0628 (14)	−0.0163 (12)	0.0155 (11)	0.0118 (12)
C19	0.0604 (14)	0.0570 (15)	0.0795 (17)	−0.0157 (12)	0.0235 (13)	−0.0025 (13)
C20	0.0643 (15)	0.091 (2)	0.0588 (15)	−0.0128 (14)	0.0202 (12)	−0.0067 (14)
C21	0.0470 (11)	0.0405 (12)	0.0429 (11)	−0.0039 (9)	0.0146 (9)	0.0012 (9)
C22	0.0580 (13)	0.0446 (12)	0.0521 (12)	−0.0020 (10)	0.0273 (10)	0.0030 (9)
C23	0.0475 (11)	0.0427 (11)	0.0398 (10)	−0.0029 (9)	0.0165 (9)	0.0011 (8)
C24	0.0701 (15)	0.0437 (12)	0.0542 (13)	−0.0003 (11)	0.0292 (11)	0.0017 (10)
C25	0.0541 (12)	0.0493 (13)	0.0549 (13)	−0.0088 (10)	0.0246 (10)	−0.0023 (10)
C26	0.0566 (13)	0.0561 (14)	0.0413 (11)	0.0072 (10)	0.0173 (10)	0.0013 (9)
C27	0.0510 (12)	0.0631 (15)	0.0548 (13)	−0.0016 (11)	0.0251 (10)	−0.0034 (11)
C28	0.0656 (14)	0.0632 (15)	0.0531 (13)	0.0123 (12)	0.0221 (11)	0.0000 (11)
C29	0.0605 (14)	0.0694 (16)	0.0560 (14)	0.0108 (12)	0.0155 (11)	−0.0096 (12)
C30	0.0701 (16)	0.0726 (17)	0.0580 (14)	0.0131 (13)	0.0196 (12)	−0.0115 (12)
C31	0.0765 (16)	0.0654 (16)	0.0512 (13)	0.0056 (13)	0.0217 (12)	−0.0117 (11)
C32	0.0639 (15)	0.0814 (18)	0.0574 (14)	−0.0151 (13)	0.0117 (12)	−0.0142 (13)

### Geometric parameters (Å, º)

**Table:**

O1—C6	1.373 (2)	C18—H18A	0.9900
O1—C31	1.418 (2)	C18—H18B	0.9900
O2—C8	1.372 (2)	C19—C20	1.507 (3)
O2—C32	1.422 (3)	C19—H19A	0.9900
O3—C11	1.226 (2)	C19—H19B	0.9900
O4—C21	1.226 (2)	C20—H20A	0.9800
C1—C3	1.361 (3)	C20—H20B	0.9800
C1—C4	1.410 (3)	C20—H20C	0.9800
C1—H1	0.9500	C21—C23	1.478 (3)
C2—C5	1.358 (3)	C22—C24	1.383 (3)
C2—C4	1.414 (3)	C22—C23	1.388 (3)
C2—H2	0.9500	C22—H22	0.9500
C3—C6	1.405 (3)	C23—C25	1.397 (3)
C3—H3	0.9500	C24—C26	1.385 (3)
C4—C7	1.426 (3)	C24—H24	0.9500
C5—C8	1.404 (3)	C25—C27	1.381 (3)
C5—H5	0.9500	C25—H25	0.9500
C6—C9	1.377 (3)	C26—C27	1.392 (3)
C7—C10	1.429 (3)	C26—C28	1.503 (3)
C7—C9	1.434 (3)	C27—H27	0.9500
C8—C10	1.379 (3)	C28—C29	1.511 (3)
C9—C11	1.510 (3)	C28—H28A	0.9900
C10—C21	1.506 (3)	C28—H28B	0.9900
C11—C14	1.480 (3)	C29—C30	1.518 (3)
C12—C13	1.380 (3)	C29—H29A	0.9900
C12—C14	1.389 (3)	C29—H29B	0.9900
C12—H12	0.9500	C30—H30A	0.9800
C13—C15	1.389 (3)	C30—H30B	0.9800
C13—H13	0.9500	C30—H30C	0.9800
C14—C16	1.394 (3)	C31—H31A	0.9800
C15—C17	1.392 (3)	C31—H31B	0.9800
C15—C18	1.511 (3)	C31—H31C	0.9800
C16—C17	1.377 (3)	C32—H32A	0.9800
C16—H16	0.9500	C32—H32B	0.9800
C17—H17	0.9500	C32—H32C	0.9800
C18—C19	1.524 (3)		
			
C6—O1—C31	118.44 (17)	C18—C19—H19A	108.7
C8—O2—C32	118.93 (18)	C20—C19—H19B	108.7
C3—C1—C4	121.7 (2)	C18—C19—H19B	108.7
C3—C1—H1	119.1	H19A—C19—H19B	107.6
C4—C1—H1	119.1	C19—C20—H20A	109.5
C5—C2—C4	121.5 (2)	C19—C20—H20B	109.5
C5—C2—H2	119.3	H20A—C20—H20B	109.5
C4—C2—H2	119.3	C19—C20—H20C	109.5
C1—C3—C6	119.0 (2)	H20A—C20—H20C	109.5
C1—C3—H3	120.5	H20B—C20—H20C	109.5
C6—C3—H3	120.5	O4—C21—C23	121.58 (18)
C1—C4—C2	120.58 (19)	O4—C21—C10	118.27 (18)
C1—C4—C7	119.70 (19)	C23—C21—C10	120.13 (17)
C2—C4—C7	119.73 (19)	C24—C22—C23	120.90 (19)
C2—C5—C8	119.1 (2)	C24—C22—H22	119.6
C2—C5—H5	120.4	C23—C22—H22	119.6
C8—C5—H5	120.4	C22—C23—C25	118.49 (19)
O1—C6—C9	115.29 (18)	C22—C23—C21	122.13 (18)
O1—C6—C3	122.58 (19)	C25—C23—C21	119.29 (18)
C9—C6—C3	122.04 (19)	C22—C24—C26	121.0 (2)
C4—C7—C10	117.79 (18)	C22—C24—H24	119.5
C4—C7—C9	117.85 (18)	C26—C24—H24	119.5
C10—C7—C9	124.36 (17)	C27—C25—C23	120.1 (2)
O2—C8—C10	114.97 (18)	C27—C25—H25	120.0
O2—C8—C5	123.04 (19)	C23—C25—H25	120.0
C10—C8—C5	121.9 (2)	C24—C26—C27	118.0 (2)
C6—C9—C7	119.69 (18)	C24—C26—C28	121.0 (2)
C6—C9—C11	117.36 (17)	C27—C26—C28	120.9 (2)
C7—C9—C11	122.22 (17)	C25—C27—C26	121.5 (2)
C8—C10—C7	119.86 (18)	C25—C27—H27	119.3
C8—C10—C21	117.30 (18)	C26—C27—H27	119.3
C7—C10—C21	122.34 (17)	C26—C28—C29	113.86 (19)
O3—C11—C14	120.92 (18)	C26—C28—H28A	108.8
O3—C11—C9	117.53 (18)	C29—C28—H28A	108.8
C14—C11—C9	121.55 (17)	C26—C28—H28B	108.8
C13—C12—C14	120.78 (19)	C29—C28—H28B	108.8
C13—C12—H12	119.6	H28A—C28—H28B	107.7
C14—C12—H12	119.6	C28—C29—C30	112.45 (19)
C12—C13—C15	121.1 (2)	C28—C29—H29A	109.1
C12—C13—H13	119.5	C30—C29—H29A	109.1
C15—C13—H13	119.5	C28—C29—H29B	109.1
C12—C14—C16	118.47 (19)	C30—C29—H29B	109.1
C12—C14—C11	122.18 (18)	H29A—C29—H29B	107.8
C16—C14—C11	119.33 (18)	C29—C30—H30A	109.5
C13—C15—C17	117.9 (2)	C29—C30—H30B	109.5
C13—C15—C18	120.1 (2)	H30A—C30—H30B	109.5
C17—C15—C18	122.0 (2)	C29—C30—H30C	109.5
C17—C16—C14	120.4 (2)	H30A—C30—H30C	109.5
C17—C16—H16	119.8	H30B—C30—H30C	109.5
C14—C16—H16	119.8	O1—C31—H31A	109.5
C16—C17—C15	121.4 (2)	O1—C31—H31B	109.5
C16—C17—H17	119.3	H31A—C31—H31B	109.5
C15—C17—H17	119.3	O1—C31—H31C	109.5
C15—C18—C19	113.14 (19)	H31A—C31—H31C	109.5
C15—C18—H18A	109.0	H31B—C31—H31C	109.5
C19—C18—H18A	109.0	O2—C32—H32A	109.5
C15—C18—H18B	109.0	O2—C32—H32B	109.5
C19—C18—H18B	109.0	H32A—C32—H32B	109.5
H18A—C18—H18B	107.8	O2—C32—H32C	109.5
C20—C19—C18	114.1 (2)	H32A—C32—H32C	109.5
C20—C19—H19A	108.7	H32B—C32—H32C	109.5
			
C4—C1—C3—C6	−0.9 (3)	C14—C12—C13—C15	−0.2 (3)
C3—C1—C4—C2	179.7 (2)	C13—C12—C14—C16	−0.9 (3)
C3—C1—C4—C7	−0.5 (3)	C13—C12—C14—C11	−179.05 (19)
C5—C2—C4—C1	179.1 (2)	O3—C11—C14—C12	−179.58 (19)
C5—C2—C4—C7	−0.8 (3)	C9—C11—C14—C12	−0.6 (3)
C4—C2—C5—C8	−1.9 (3)	O3—C11—C14—C16	2.3 (3)
C31—O1—C6—C9	173.89 (19)	C9—C11—C14—C16	−178.71 (17)
C31—O1—C6—C3	−9.4 (3)	C12—C13—C15—C17	1.2 (3)
C1—C3—C6—O1	−175.9 (2)	C12—C13—C15—C18	−178.8 (2)
C1—C3—C6—C9	0.6 (3)	C12—C14—C16—C17	1.0 (3)
C1—C4—C7—C10	−177.32 (19)	C11—C14—C16—C17	179.17 (18)
C2—C4—C7—C10	2.5 (3)	C14—C16—C17—C15	0.1 (3)
C1—C4—C7—C9	2.0 (3)	C13—C15—C17—C16	−1.2 (3)
C2—C4—C7—C9	−178.17 (18)	C18—C15—C17—C16	178.9 (2)
C32—O2—C8—C10	165.2 (2)	C13—C15—C18—C19	68.8 (3)
C32—O2—C8—C5	−18.2 (3)	C17—C15—C18—C19	−111.2 (3)
C2—C5—C8—O2	−173.6 (2)	C15—C18—C19—C20	62.7 (3)
C2—C5—C8—C10	2.8 (3)	C8—C10—C21—O4	107.1 (2)
O1—C6—C9—C7	177.76 (17)	C7—C10—C21—O4	−64.8 (3)
C3—C6—C9—C7	1.0 (3)	C8—C10—C21—C23	−74.3 (2)
O1—C6—C9—C11	7.3 (3)	C7—C10—C21—C23	113.7 (2)
C3—C6—C9—C11	−169.44 (19)	C24—C22—C23—C25	0.0 (3)
C4—C7—C9—C6	−2.2 (3)	C24—C22—C23—C21	−176.60 (19)
C10—C7—C9—C6	177.02 (18)	O4—C21—C23—C22	−178.17 (19)
C4—C7—C9—C11	167.69 (17)	C10—C21—C23—C22	3.3 (3)
C10—C7—C9—C11	−13.1 (3)	O4—C21—C23—C25	5.2 (3)
O2—C8—C10—C7	175.68 (17)	C10—C21—C23—C25	−173.28 (18)
C5—C8—C10—C7	−1.0 (3)	C23—C22—C24—C26	1.1 (3)
O2—C8—C10—C21	3.5 (3)	C22—C23—C25—C27	−0.6 (3)
C5—C8—C10—C21	−173.08 (19)	C21—C23—C25—C27	176.10 (19)
C4—C7—C10—C8	−1.7 (3)	C22—C24—C26—C27	−1.7 (3)
C9—C7—C10—C8	179.06 (19)	C22—C24—C26—C28	178.9 (2)
C4—C7—C10—C21	170.04 (18)	C23—C25—C27—C26	0.1 (3)
C9—C7—C10—C21	−9.2 (3)	C24—C26—C27—C25	1.1 (3)
C6—C9—C11—O3	106.1 (2)	C28—C26—C27—C25	−179.5 (2)
C7—C9—C11—O3	−64.1 (3)	C24—C26—C28—C29	94.9 (3)
C6—C9—C11—C14	−73.0 (2)	C27—C26—C28—C29	−84.5 (3)
C7—C9—C11—C14	116.9 (2)	C26—C28—C29—C30	177.9 (2)

### Hydrogen-bond geometry (Å, º)

**Table:**

*D*—H···*A*	*D*—H	H···*A*	*D*···*A*	*D*—H···*A*
C13—H13···O3^i^	0.95	2.41	3.342 (3)	168
C24—H24···O4^ii^	0.95	2.45	3.390 (3)	170

Symmetry codes: (i) *x*, *y*+1, *z*; (ii) *x*, *y*−1, *z*.
